# Close to me but unreachable: spotting the link between peripersonal space and empathy

**DOI:** 10.1093/scan/nsad030

**Published:** 2023-05-27

**Authors:** Arianna Schiano Lomoriello, Chiara Cantoni, Pier Francesco Ferrari, Paola Sessa

**Affiliations:** Section for Cognitive Systems, DTU Compute, Technical University of Denmark, Kgs. Lyngby 2800, Denmark; Department of Psychology, Sapienza University of Rome, Rome 00185, Italy; IRCCS, Santa Lucia Foundation, Rome 00142, Italy; Institut des Sciences Cognitives Marc Jeannerod, Unit 5229, CNRS/Université Claude Bernard Lyon, Bron Cedex 69675, France; Department of Developmental and Social Psychology, University of Padova, Padova 35121, Italy; Padova Neuroscience Center (PNC), University of Padova, Padova 35121, Italy

**Keywords:** peripersonal space, empathy, plexiglass, barrier, embodiment, COVID-19

## Abstract

The space surrounding the body [i.e. peripersonal space (PPS)] has a crucial impact on individuals’ interactions with the environment. Research showed that the interaction within the PPS increases individuals’ behavioral and neural responses. Furthermore, individuals’ empathy is affected by the distance between them and the observed stimuli. This study investigated empathic responses to painfully stimulated or gently touched faces presented within the PPS depending on the presence *vs* absence of a transparent barrier erected to prevent the interaction. To this aim, participants had to determine whether faces were painfully stimulated or gently touched, while their electroencephalographic signals were recorded. Brain activity [i.e. event-related potentials (ERPs) and source activations] was separately compared for the two types of stimuli (i.e. gently touched *vs* painfully stimulated faces) across two barrier conditions: (i) no-barrier between participants and the screen (i.e. no-barrier) and (ii) a plexiglass barrier erected between participants and the screen (i.e. barrier). While the barrier did not affect performance behaviorally, it reduced cortical activation at both the ERP and source activation levels in brain areas that regulate the interpersonal interaction (i.e. primary, somatosensory, premotor cortices and inferior frontal gyrus). These findings suggest that the barrier, precluding the possibility of interacting, reduced the observer’s empathy.

## Introduction

The space around the body is fundamentally important for individuals’ interactions with objects and others. This area, known as the peripersonal space (PPS), is the multisensory interface between the body and the environment. Within the PPS, objects are within arm’s reach and are coded in terms of potential action (Rizzolatti *et al.*, 1997; Previc, 1998; Berti and Frassinetti, 2000; Holmes and Spence, 2004; Coello and Delevoye-Turrell, 2007; Cardellicchio *et al.*, 2011; Iachini *et al.*, 2014; Wamain *et al.*, 2016).

In the present study, we aimed at investigating the link between the PPS and neural empathic reactions. To this aim, we manipulated the PPS by means of a transparent plexiglass barrier placed between the observer and the target of their empathy.

While the PPS is indeed commonly considered a discrete, distance-based, in-or-out space, some behavioral studies have contradicted this view, suggesting that the PPS lacks a sharp spatial boundary (Bufacchi and Iannetti, 2018). In particular, PPS-related neurons in animals have been shown to respond to stimuli with a graded relationship to distance (Duhamel *et al.*, 1998). For instance, studies of single bimodal neurons in macaques have documented larger activation in cortical and subcortical structures (i.e. putamen, parietal and premotor areas) in response to visual or auditory stimuli presented in spatial proximity (Colby *et al.*, 1993; Graziano and Gross, 1993; Ladavas *et al.*, 2001). However, research has shown that this effect is abolished when visual stimuli are presented beyond arm’s reach (e.g. Rizzolatti *et al.*, 1981; Fogassi *et al.*, 1999; Graziano and Gross, 1993), in the so-called extrapersonal space. To note, the extrapersonal space is determined not only by arm’s reach but also by the brain’s representation of the extended body space (Berti and Frassinetti, 2000), the nature of the stimuli and the type of interactions envisaged (Geers and Coello, 2023). Human behavioral and neural studies [see, e.g., Maravita *et al.* (2003), Longo and Lourenco (2007) and Macaluso and Maravita (2010) for reviews] have also documented distance-dependent modulations of processes within and beyond the PPS, reporting increased activation in sensorimotor brain areas in response to manipulable objects located within the PPS. This effect has been explained as a consequence of the motor nature of this space [i.e. see Culham *et al.*, (2008); Proverbio (2012) and Wamain *et al.* (2016) for perceptual stimuli; see Wamain *et al.* (2018) for semantic stimuli and see Coventry *et al.* (2008) and Coello and Bonnotte (2013) for conceptual information about objects]. Of note, distance from the object is not the only relevant factor. Caggiano *et al.* (2009) found that the mirror neurons in rhesus monkeys presented different activation patterns in response to observed action in the PPS *vs* the extrapersonal space, depending on whether a transparent barrier was present that prevented the monkeys from touching the nearby objects. The authors reported that approximately half of the tested space–selective mirror neurons were influenced by the presence of the panel, whereby extrapersonal-sensitive space neurons started to respond in the PPS, while PPS-sensitive space neurons ceased to respond. This finding was confirmed in a study on monkeys by Bonini *et al.* (2014) in which it was found that canonical-mirror neurons in F5 discharged weakly to the presentation of an object when this occurred behind a transparent plastic barrier. Critically, introducing a transparent barrier did not change the metric distance between the monkey and the object, but it did change the operational space. This suggests that the PPS and extrapersonal space are dynamic, receptive fields that are neuronally defined according to the possibility for action, rather than metric distance, thus providing a pragmatic encoding of objects in space.

Building on this idea, some evidence suggests that the PPS not only allows for motor engagement with objects but also mediates possible interactions within the space (Heed *et al.*, 2010; Di Pellegrino and Làdavas, 2015). For example, Duhamel *et al.* (1998) found increased behavioral responses when individuals interacted in a space defined by an arm’s length—a phenomenon that social psychologists have related to the evolutionary principle that a person within striking distance is more relevant than a person positioned farther away (van der Stoep *et al.*, 2015). Interestingly, at the neural level, functional magnetic resonance imaging (fMRI) studies have documented that the amygdala is differentially activated based on proximity to another person, showing greater activation under conditions of close personal proximity (Kennedy *et al.*, 2009; Schienle *et al.*, 2015).

Along this line, Heed *et al.* (2010) administered the classical visuo-tactile cross-modal congruency task (e.g. Pavani *et al.*, 2000; Spence *et al.*, 2004; Holmes, 2012), requiring participants to respond to the elevation of a vibrotactile target delivered to the index or thumb of either hand, while ignoring a simultaneous visual distractor. Participants who performed the task with another person showed reduced cross-modal interference in tactile judgment, but only when the partner was physically situated within the participant’s PPS. This result can be explained as a top-down modulation of multisensory integration, whereby the representation of the partner’s task changed the relative contributions of the visual and tactile modalities to tactile judgments.

Some preliminary evidence (while sparse) also indicates a link between the spatial PPS representation and empathy. For example, Boukricha *et al.* (2011) found that PPS representations changed according to inter-individual differences, whereby more empathetic individuals were more likely to share their PPS with another individual during a cooperative task; this suggests a positive relationship between empathy and the physical interaction. Furthermore, Mahayana *et al.* (2014) demonstrated that participants showed pain empathic responses when viewing pictures of others’ body parts in painful situations only when the pictures were presented within the PPS, and not when the pictures were presented in the extrapersonal space. Of note, an event-related potential (ERP) study corroborated this finding by demonstrating that perceived physical distance between individuals tended to shape their empathic reactions toward others in pain (Schiano Lomoriello *et al.*, 2018). In this study, the authors found a modulation of ERP amplitude (in the time window corresponding to the ERP P3 component) as a function of the perceived distance between the observer and the observed faces. The ERP amplitude in late time windows, reflecting a more cognitive aspect of empathy (i.e. mentalizing), was larger for faces perceived as closer than for those perceived as more distant, suggesting a stronger empathic reaction to individuals perceived as nearer to the observer. Among the interpretations provided by the authors of the study to explain their results, they also referred to the Embodied Cognition theories, whereby most cognitive processes depend on, reflect, or are influenced by the body’s control systems (e.g. Caruana and Borghi, 2013). The authors recalled the concept of embodiment known as embodied simulation (Gallese, 2005), which is a functional mechanism that allows individuals to understand the meaning of actions and emotions of others. Embodied simulation is linked to intersubjectivity, specifically mirroring, which means that the same neural mechanisms are activated when an individual experiences similar emotions and sensations as others (e.g. Gallese, 2010). According to this framework, embodied simulation and mirroring are thought to underlie the more automatic component of empathy (Gallese and Goldman, 1998; Gallese, 2003, 2008; Gallese *et al.*, 2006; Singer and Lamm, 2009; Lamm and Singer, 2010; Uithol *et al.*, 2011). Interestingly, the above-mentioned findings propose the idea that the embodied simulation mechanism is sensitive to physical distance and interactive space between two individuals, suggesting a link between empathy and the spatial representation of the PPS.

To date, no study has investigated whether individuals’ empathic responses are affected when the interaction is impeded, even when both persons are sharing the same interactive space. In the present study, we specifically investigated the link between the PPS and empathy by testing whether brain activity (i.e. ERPs and source activations) connected to the ability to empathize and interact with others was dampened when a transparent plexiglass barrier was placed between the observer and the observed stimuli, within the observer’s PPS, without otherwise altering the quality or low-level features of the stimuli. Participants were asked to judge whether faces were being gently touched by a Q-tip or painfully stimulated by a syringe in two critical experimental conditions (i.e. no-barrier *vs* barrier) in a within-subject design, while we recorded their electrical activity. At the neuroanatomical level, empathy can be differentiated into experience-sharing mechanisms (likely involving mirror neurons), the limbic system and mentalizing (involving prefrontal and temporal cortex regions and the precuneus) (Amodio and Frith, 2006; Shamay-Tsoory *et al.*, 2009; Betti and Aglioti, 2016). This differentiation was also evident in electrical brain activity, as ERPs showed amplitude modulations related to the pain condition processing of the early, experience-sharing component (0–300 ms; N1, P2 and N2–N3) and later, mentalizing components (300–650 ms; P3) in the pain decision task, manifesting as a positive shift in the painful condition relative to the neutral condition [Fan and Han, 2008; see also, e.g., Meconi *et al.*, 2018; Sessa *et al.*, 2014; see also Zaki and Ochsner (2012) for a review on this topic]. More generally, an empathic reaction at the ERP level manifests itself as a positive shift in neural activity with respect to a control condition toward which the positive shift is quantified.

Of note, the ability to understand another person’s experience is fundamental for social interactions and subserved by the same neural structures as those involved in first-person experience (of, e.g., pain) (Preston and de Waal, 2002). The sensory discriminative aspects of observed pain are associated with activity in the primary (S1) and secondary (S2) somatosensory cortices (Bufalari *et al.*, 2007; Saarela *et al.*, 2007; Costantini *et al.*, 2008; Valeriani *et al.*, 2008; Akitsuki and Decety, 2009; Betti *et al.*, 2009; Voisin *et al.*, 2011; Aziz-Zadeh *et al.*, 2012), as well as in the primary motor cortex (M1) (Avenanti *et al.*, 2005). Thus, empathic responses in these regions may reflect a process that represents bodily and affective states originating in both the self and others, with the aim of guiding behavioral responses (Singer and Lamm, 2009). Since the present study aimed at highlighting modulations of empathic reactions as a function of a plexiglass barrier between an observer and an observed face, two methodological/analytical choices were made. First, all of the presented results emerged from a comparison between the brain activity (ERPs and source activations) elicited when the plexiglass was present *vs* when it was absent, for the two stimulation conditions (i.e. gentle touch *vs* pain), separately. Second, to manage the multiple comparison problem and the risk of type-I error—which are particularly relevant to large, spatio-temporal datasets such as those produced by the electroencephalographic (EEG) research—the state-of-the-art cluster-based permutation approach was used, considering two classical and dissociable temporal windows associated with the well-known and above-mentioned aspects of empathy: experience sharing and mentalizing (Maris and Oostenveld, 2007).

Using the contrastive approach described earlier (i.e. barrier *vs* no-barrier), we expected a reduction in both experience sharing and mentalizing ERP components (i.e. a negative shift in brain electrical activity relative to a baseline) toward gently touched and painfully stimulated faces in the barrier *vs* the no-barrier condition. Furthermore, at the source level, due to the alteration to the PPS caused by the presence of the barrier, we expected attenuated brain activity in those regions crucial for embodied simulation [e.g. premotor cortex and inferior frontal gyrus (IFG)], reflecting both the observer’s inability to reach the other person (and consequently to share the other’s sensory state) and the modulation of his/her resonance mechanisms.

## Method

### Participants

Data were collected from 30 volunteer healthy students (7 male) from the University of Padova. Data from five participants were excluded from the analyses due to excessive electrophysiological artifacts. All participants reported normal or corrected-to-normal vision, normal audition and no history of neurological disorder. The final sample included 25 participants (4 male; *M*_age_ = 20.4 years, s.d. = 1.93, 1 left-handed), in line with a reference study (Schiano Lomoriello *et al.*, 2018). A power analysis using data simulation for cluster-based permutation tests (Wang and Zhang, 2021) revealed that a sample size of 24 participants is sufficient to obtain the power of at least 80% when detecting differences in ERP data between two conditions (i.e. barrier *vs* no-barrier) in a within-subject design. All participants signed a consent form, in line with the ethical principles approved by the University of Padova (protocol no. 1185).

### Stimuli

The stimuli were 12 digital photographs of white faces with neutral facial expressions from the Eberhardt Lab Face (ELF) database (Mind, Culture, & Society Laboratory, Stanford University). Each face was digitally manipulated to obtain static images for two conditions: one in which faces were receiving a painful stimulation (insertion of a syringe) on the left or right cheek and one in which faces were being gently touched with a Q-tip on the left or right cheek. All faces were presented in an upright orientation in the dimensions 2.5° × 3.3° (width × height). Stimuli were presented on a 17-inch cathode ray tube monitor controlled by a computer running E-prime software.

### Procedure

**Fig. 1.**
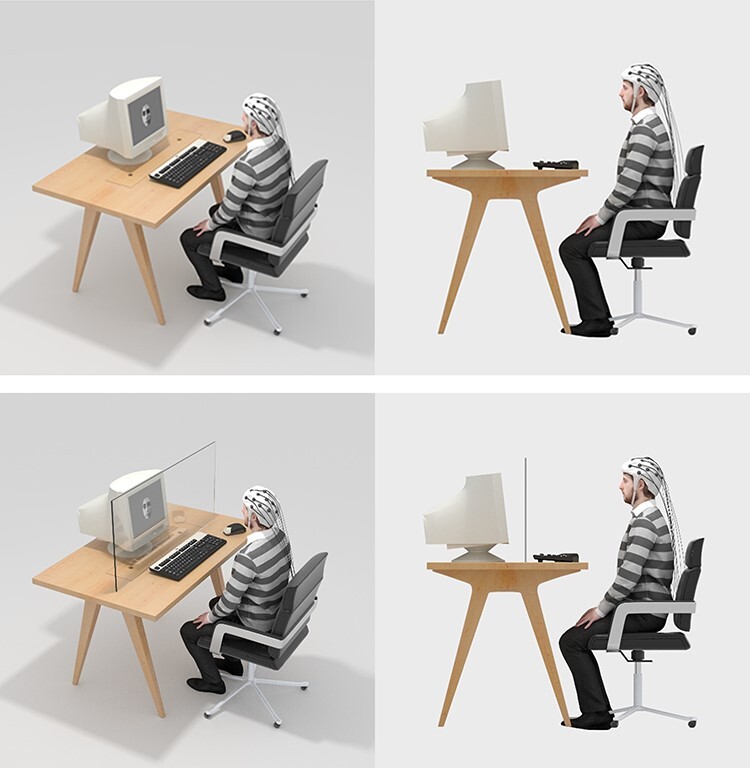
Experimental setup, including the no-barrier condition (top) and the barrier condition (bottom).

**Fig. 2.**
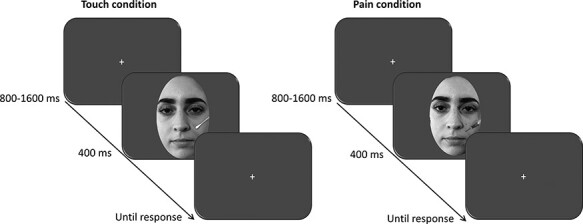
The timeline of each trial in touch (left) and pain (right) panels. Original face stimuli have been replaced with actors according to the terms of use of the ELF database.

The present study implemented a stimulation discrimination task. Each trial began with the presentation of a fixation cross at the center of the screen (800–1600 ms, jittered in steps of 100 ms), followed by a face, which was displayed for 400 ms. Figure 1 depicts the sequence of events in each trial. Participants were instructed to differentiate between faces being gently touched by a Q-tip and faces being painfully stimulated (intermixed within the experiment) by pressing one of two appropriately labeled keys on the computer keyboard as accurately as possible. Half of the participants were instructed to press the ‘F’ key to indicate that the face was being gently touched by a Q-tip and the ‘J’ key to indicate that the face was being painfully stimulated. The other half of the participants were instructed to register their responses according to the inverse pattern. No time pressure conditions were applied, and participants were informed that the speed of their responses would not be considered in the evaluation of their performance. To test our hypothesis, each participant performed the task in two critical conditions (with all participants following a counterbalanced order): in the barrier condition, a transparent panel was interposed between each participant and the computer monitor, to interfere with individuals’ PPS and in the no-barrier condition, nothing was interposed between the participant and the computer monitor (Figure 1). The plexiglass barrier was a transparent 100 × 70 × 0.8 cm (width × height × thickness) poly (methyl methacrylate) screen positioned ∼40 cm from the participant’s face and 30 cm from the monitor. The experiment started with a block of 12 practice trials, so that participants could familiarize themselves with the task. Participants completed the actual task in two sessions of 384 trials, each. Each session was divided into 6 blocks (with 192 trials in each block), and participants could take a break between blocks and self-elect when to continue by pressing the space bar (Figure 2). Each session lasted ∼15 min. The entire experimental session, including the preparation of the participant for the EEG data collection, lasted ∼40 min.

#### EEG data preprocessing

EEG data were collected and recorded by means of 64 active electrodes, which were distributed on participants’ scalp according to the extended 10/20 system, with an elastic actiCAP positioned with reference to the left ear lobe. The EEG was re-referenced offline to the average of the left and right earlobes. The horizontal EOG (i.e. HEOG) was recorded bipolarly from two external electrodes positioned laterally to the left and right external canthi. The vertical EOG (i.e. VEOG) was recorded from Fp1 and one external electrode placed below the left eye. The electrode impedance was kept <10 KΩ, due to the highly viscous electro-gel and the properties of active electrodes. Offline EEG processing and analyses were conducted using the BrainVision Analyzer software (Brain Products). The sampling rate was set to 1000 Hz. Continuous data were down-sampled to 500 Hz, high-pass filtered at 0.1 Hz, re-referenced to the average of all channels and segmented in epochs from −100 to 1000 ms, with respect to the stimulus onset. Independent component analysis was applied to the segmented data to identify and manually remove artifactual activity related to eye-blinks and saccades (Jung *et al.*, 2000). Separate average waveforms for each condition were then time-locked to the presentation of the face stimuli as a function of the preceding context.

Early (0–350 ms) and late (350–650 ms) time windows were considered, in line with evidence on the dissociability between an early and late ERP empathy–related response, i.e. experience sharing and mentalization, respectively (Sessa *et al.*, 2014; Meconi *et al.*, 2018; Palmieri *et al.*, 2021).

In the ‘Results’ section, we refer to 0–350 ms (experience sharing) and 350–650 ms (mentalizing) time windows, instead of empathic components, to help the reader better understand the results.

#### Statistical analysis

To test our behavioral hypothesis (i.e. that the presence of a transparent panel would modulate empathic reactions toward faces being painfully stimulated and faces being gently touched with a Q-tip), we employed linear mixed-effect (LME) modeling. Specifically, LME models were applied separately to the behavioral data (referring to accuracy and reaction time) and the ERP components. In each model, we included as fixed effects the stimulation (pain *vs* touch), the condition (barrier *vs* no-barrier) and the interaction between them. The full model structure for both accuracy and ERP measures in the Wilkinson notation was dependent variable∼stimulation * condition + (1|ID). The random effect structure included participants as random intercepts, thereby adjusting for individual differences in the dependent variable.

Starting with the full model (i.e. including all interactions between predictors), we identified the combination of predictors that best described the data using a stepwise approach based on the Akaike information criterion (AIC) model selection strategy (Wagenmakers and Farrell, 2004). The use of the AIC (Akaike, 1973) is a well-established data-driven procedure for selecting the best combination of parameters to fit the data, considering that an under-fitted model may not capture the true variability of the outcome variable, while an over-fitted model will lack generality. The AIC strategy compares models on a given outcome and selects the model that best represents the true relationship with the given data. Mixed models are compared on the basis of the −2 (restricted) log likelihood of information theory, as a measure of relative quality. The model with the lowest AIC value is considered the best-fitting model (i.e. representing the optimal trade-off between goodness of fit and parsimony, in terms of the number of parameters) (Burnham *et al.*, 2011). This strategy has been widely applied in various research fields and with different types of data (e.g. ERPs: Hall *et al.*, 2006; Schiano Lomoriello *et al.*, 2021; behavioral: Novick *et al.*, 2013; Boldrini *et al.*, 2020).

In the present study, the best-fitting LME models were used for further analyses. All analyses were conducted using the R software (4.2), specifically the lmer function from the lme4 package (Bates *et al.*, 2015). Significance levels for fixed and random effects were computed using the anova function in the lmerTest package, which applies Satterthwaite’s approximation for degrees of freedom. *Post hoc* comparisons were computed using the PHIA package (i.e. *post hoc* interaction analysis), corrected for multiple comparisons using the false discovery rate (Benjamini and Hochberg, 1995).

All datasets and analyses are available within the Open Science Framework (OSF) repository: https://osf.io/6uxgt/.

#### EEG statistical analysis

To manage multiple comparisons and type-I error, we applied the state-of-the-art cluster permutation *t*-tests (Bullmore *et al.*, 1999; Maris and Oostenveld, 2007), using Brainstorm (Tadel *et al.*, 2011), as performed in precedent studies (e.g., Sessa *et al.*, 2022; Schiano Lomoriello *et al.*, 2022). Specifically, we conducted a whole-scalp analysis across all 64 electrode sites in the 0–350 ms time window, using a paired *t*-test cluster permutation approach (cluster α = 0.05, 5000 within-participant random permutations of the data) to control for the family-wise error rate (Groppe *et al.*, 2011). In doing so, we used the FieldTrip function (Maris and Oostenveld, 2007) within Brainstorm (Tadel *et al.*, 2011).

#### Cortical source modeling

Although electrophysiological techniques are limited in their spatial resolution, some studies have demonstrated that it is possible to investigate the temporal dynamics of reconstructed cortical activity using brain source analysis with 64 channels (Hassan *et al.*, 2014). In the present study, baseline-corrected epochs were imported into Brainstorm (Tadel *et al.*, 2011) for the modeling of cortical generators. Using the ICBM152 anatomical template, we approximated the individual anatomy of each participant (Evans *et al.*, 2012). Co-registration of the EEG electrode position was performed in Brainstorm, by projecting the digitized EEG sensor positions of the BrainProducts actiCAP 65 (available in Brainstorm) onto the head surface. We then derived an EEG forward model using the three-layer boundary element method from OpenMEEG, implemented as a Brainstorm routine (Kybic *et al.*, 2005; Gramfort *et al.*, 2011). The source space was constrained to the cortex and modeled as a grid of 15.002 orthogonal current dipole triplets. We used sLORETA as a source model, with Brainstorm’s default parameter settings. The empirical noise covariance model was obtained from the average ERP baseline signals. Sources were projected to the standard anatomical template Montreal Neurological Institute and their activity was transformed into *z*-scores relative to the baseline. Finally, a spatial smooth with a 3 mm Full width at half maximum was applied to each source.

## Results

### Behavioral

**Fig. 3.**
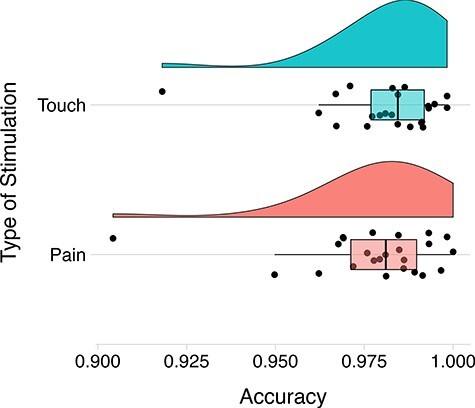
Accuracy for faces being gently touched by a Q-tip (top panel) and faces being painfully stimulated (bottom panel). The boxplots represent the minimum, maximum, lower and upper quartiles and median. The dots represent participant responses.

From the LME model applied to participant accuracy, the AIC model comparison showed that the model that best explained the data was that which included the stimulation, the condition and the interaction between them as fixed effects, with a random intercept to model repeated measurements across participants (AIC = 5248.5, log*L* = −2669.261, ΔAIC^1^ = -107.4). Thus, participant accuracy was regressed on these sets of regressors [i.e. in the Wilkinson notation: accuracy∼condition + stimulation + condition: stimulation + (1|ID)], and significance levels were computed for the fixed and random effects, using the anova function (lmerTest package), which returned a type-III ANOVA table (Wald χ^2^ tests) with significance levels. A main effect of the stimulation [χ^2^ (1, *N *= 25) = 0.0313, *P* = 0.014] was found, indicating that participants’ performance was higher when they observed faces being gently touched by a Q-tip (*M*_scores_ = 0.982) rather than faces being painfully stimulated (*M*_scores_ = 0.971) [χ^2^ (1, *N *= 25) = 4.165, *P* = 0.004; *M*_diff_ = 0.004 (0.003–0.37)] (Figure 3). Neither a condition effect nor an interaction between the stimulation and the condition was found. No effect was found for response speed; however, this factor was not stressed in the instructions given to participants (min *P* = 0.59).

### Electroencephalography

**Fig. 4.**
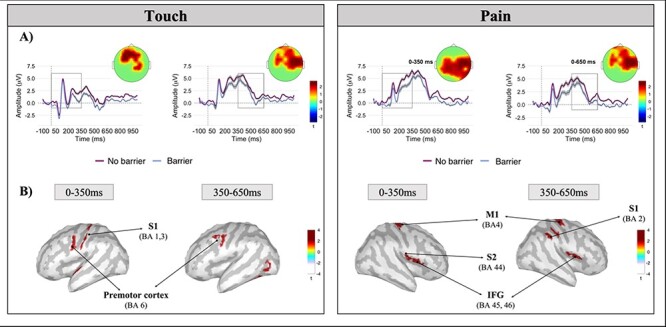
(A) The panel displays the grand averages of the ERPs at cluster sites in each experimental condition. Topographies are shown for each time window (the left topographies show the scalp distribution of the averaged activity in the 0–350 and 350–650 time windows corresponding to experience sharing and metalizing, respectively, for the touch condition; the right topographies show the scalp distribution of the averaged activity in the 0–350 and 350–650 time windows for the pain condition). The red dots inside the activated areas (in yellow) represent sites that formed a cluster; ERPs are plotted as the average of the significant cluster activity. Shades represent the confidence intervals. (B) The panel presents the statistical difference in the source map between the no-barrier and barrier conditions, following the presentation of the stimulus, separated for the type of stimulation: gentle touch (on the left) and pain (on the right). Significant clusters (*P* < 0.05) are reported on a template cortex smoothed at 100%. The right panel shows more significant activity for faces being gently touched by a Q-tip when observed in the no-barrier condition [time window 0–350 ms: primary somatosensory cortex (S1; Brodmann areas 1 and 3) and premotor cortex (Brodmann area 6); time window 350–365 ms: premotor cortex (Brodmann area 6)]. The left panel shows the greater activity for faces being painfully stimulated [time window 0–350 ms: motor cortex (MI; Brodmann area 4), secondary somatosensory cortex (S2; Brodmann area 44) and IFG [Brodmann areas 45 and 46]; time window 350–650 ms: S1, M1 and IFG].

Figure 4A shows the grand average ERP component, time-locked to the onset of the face, as a function of the sites that formed a cluster. Each experimental condition is presented with the ERPs that were elicited by observing the painful and gentle stimulation, separately, with and without the plexiglass barrier. The topographies of each graph represent scalp activity in the respective time window.

Cluster permutation *t*-test analysis in the first time window (0–350 ms) revealed a significant difference in participants’ empathic neural responses between the no-barrier condition when faces were being painfully stimulated (positive cluster: *P*_corr_ = 0.002; cluster size = 62, cluster statistic = 91) or gently touched by a Q-tip (positive cluster: *P*_corr_ = 0.014; cluster size = 24, cluster statistic = 33) and the barrier condition. A significant difference was also found in the subsequent time window (i.e. 350–650 ms) for the no-barrier condition when faces were gently touched by a Q-tip (positive cluster: *P*_corr_ = 0.002; cluster size = 46, cluster statistic = 92, *P*_corr_ = 0.044) or painfully stimulated (positive cluster: *P*_corr_ = 0.04; cluster size = 46, cluster statistic = 52), relative to the barrier condition (Figure 4).

To assess whether the presence of a transparent barrier might have altered participants’ cortical activity when observing faces being painfully stimulated and gently touched by a Q-tip, respectively, we performed one-tailed permutations at the source level in the two differently averaged time windows (i.e. 0–350 and 350–650 ms; *P* < 0.05). In the 0–350 ms time window for faces being gently touched, source statistics revealed higher activity in the primary somatosensory (S1) and premotor cortices in the no-barrier condition compared to the barrier condition. In the 350–650 ms time window, only the premotor cortices maintained a similar level of activation (Figure 4B, left panel). In the 0–350 ms window for faces being painfully touched, the no-barrier condition demonstrated stronger activation in the primary motor cortex (M1), the secondary somatosensory cortex (S2) and the IFG. The IFG also remained more active in the subsequent time window, together with the S1 (Figure 4B, right panel).

## Discussion

The present investigation focused on the relation between the PPS and empathy by directly interfering with participants’ PPS as they observed faces being either painfully pierced by a syringe or gently touched with a Q-tip. The experimental manipulation required that, in half of the trials, a transparent panel was interposed between the participant and the stimuli, preventing the potential interaction; in the other half of the trials, no-barrier was present. Critically, the stimuli were always within arm’s reach of the observer (Nguyen and Wachsmuth, 2011). The findings revealed a close relationship between the PPS and empathy, as the presence of a barrier within the PPS that separated participants from the stimuli (without impeding their vision) tended to impact their empathic responses.

At the ERP level, we observed a reduction in amplitude along the entire time course from stimulus onset, regardless of the type of facial stimulation. It has been suggested that the PPS may serve as a buffer with respect to the spatial adjustments required by social interactions [see Coello and Cartaud (2021) for a review]. As we had no a priori hypothesis regarding the possible temporal and spatial distributions of the effects, we adopted a non-parametric permutative approach, considering two classical and dissociable temporal windows associated with the well-known aspects of empathy, which include cognitive and somatomotor components (Davis, 1996; Preston and de Waal, 2002; Gallese, 2003; Decety and Jackson, 2004; Avenanti and Aglioti, 2006). Previous ERP studies have shown that empathic responses are characterized by a positive shift in brain electrical activity compared to a baseline condition over a 600–800 ms time interval following the presentation of a stimulus [Sessa *et al.*, 2014; Palmieri *et al.*, 2020; see also Donchin, 1981; Donchin and Coles, 1988; Verleger, 1988; Sessa *et al.*, 2007; Sessa and Meconi, 2015; Sheng *et al.*, 2015; Schiano Lomoriello *et al.*, 2018; see also Coll (2018) for a methodological review]. According to this, the plexiglass seems to have inhibited both the early and later components of the empathic response.

Along the same lines, we found significantly higher activation in motor, premotor and somatosensory areas in the no-barrier condition. Interestingly, these regions are known to be involved in the process of mapping others’ sensations onto one’s own sensorimotor system, thereby connecting with others (Hennenlotter *et al.*, 2005; Warren *et al.*, 2006; Gazzola and Keysers, 2009; Balconi and Bortolotti, 2012). As demonstrated in rhesus monkey by Caggiano, a portion of mirror neurons encode space according to a metric representation, whereas other neurons encode space in operational terms, changing their properties according to the possibility that the monkey will interact with the object. These sites are also involved in determining the correct interpersonal response to a given situation (Caggiano *et al.*, 2009). The reduced activation found in the present study can be explained as a consequence of the decrease in empathic response due to disengagement in the interaction and the inability to potentially physically interact.

Previous research has found that activation in the somatosensory regions is positively linked with empathy (Keysers and Gazzola, 2006; Keysers *et al.*, 2010); thus, it is reasonable to suppose that less activation in these areas may reflect a decrease in neural empathic response. In a meta-analysis of nine fMRI experiments, Lamm *et al.* (2011) found that vicarious activation of the somatosensory cortex occurs only when visual details of the painful situation are observed, and not when these are inferred from abstract cues. The authors argued that this activation reflects non-specific co-activation elicited by the visualization of body parts, rather than a specific correspondence between the somatosensory and nociceptive states, in line with the characterization of empathy as, first and foremost, an affective state (Gallese and Goldman, 1998; Gallese, 2003, 2008; Gallese *et al.*, 2006; Csibra, 2008; Hickok, 2009; Singer and Lamm, 2009; Lamm and Singer, 2010; Uithol *et al.*, 2011; see also Lamm and Majdandžić, 2015). Other authors have argued for the functional importance of primary sensory cortices as part of the empathic response, as such cortices are involved in encoding the intensity and location of pain (Keysers *et al.*, 2010). Osborn and Derbyshire (2010) reported that individuals who respond to painful images by experiencing a ‘real’ sensation of pain show activation in the somatosensory cortices, while individuals who lack these direct experiences do not demonstrate the equivalent activation. Further evidence for the involvement of sensory cortices in the observation of pain comes from EEG studies. Bufalari *et al.* (2007) showed participants’ video clips depicting people in painful situations involving their limbs, recording decreased early sensory–evoked potentials following medial nerve stimulation. Several studies have also demonstrated a significant relation between pain systems and action systems (Ingvar, 1999; Saitoh *et al.*, 1999; Juottonen *et al.*, 2002; Farina *et al.*, 2003; Wager *et al.*, 2004). For instance, Avenanti *et al.* (2005, 2006, 2009) showed that repeated viewing of video clips depicting pain significantly inhibits the muscle-specific corticospinal excitability that is typically observed during pinching. Somatosensory neural structures may also impact representations of touch. In fact, some studies (Keysers *et al.*, 2004; Blakemore *et al.*, 2005) have shown that the observation of individuals receiving tactile stimulation induces activity in somatosensory cortices—areas that are typically involved in the sensation of touch and therefore the experience of pain (Porro *et al.*, 1998; Ploner *et al.*, 2000; Timmermann *et al.*, 2001; Bingel *et al.*, 2004). These findings suggest that somatosensory regions are highly relevant to the empathic response, over and above their role in indicating a non-specific increase in arousal.

It is reasonable to explain the lower activation found in the motor and somatosensory areas as a drop in the participants’ empathic response due to the impediment of the plexiglass, which made it impossible for participants to potentially physically interact with the stimuli. In this regard, it is important to note that the mirror neuron system, in addition to encoding and observing motor acts, also contributes to the selection of appropriate behavioral responses and empathy. It has been suggested that people are able to understand and share the emotions of others by processing them (partially) through their own emotional system. This effect is known as mirroring, which has been linked to empathy (Gallese and Goldman, 1998). Thus, it is reasonable to assume that reduced brain activity in these areas may reflect decreased empathic response, over and above the inability to reach out to the other. In fact, mirror neurons seem to encode space in operational (rather than metric) terms, thereby modifying their properties according to behavioral contingencies, such as the possibility or impossibility of the physical interaction. On this basis, it seems that mirror neurons play a cognitive role, representing a neuronal substrate for understanding the actions of others and determining appropriate interpersonal behavior in response to these actions. For example, Wamain *et al.* (2016) found a desynchronization of the μ rhythm (which has been widely associated with motor preparation and execution; Salmelin and Hari, 1994; Salenius *et al.*, 1997; Babiloni *et al.*, 1999; Llanos *et al.*, 2013) in the centro–parietal EEG activity of healthy adults when objects were placed within the PPS, with the effect progressively decreasing as objects were moved toward and into the extrapersonal space. Likewise, Cardellicchio *et al.* (2011) observed higher motor-evoked potentials when participants observed graspable objects within the PPS rather than ungraspable or graspable objects outside the PPS. It should be added that the lower activation found in the premotor cortices may not necessarily reflect a decrease in mirror neuron activity. Previous studies conducted on macaques have found that neurons in the caudal part of F4 are somatotopically organized, demonstrating that the face is the most representative part of the body (Gentilucci *et al.*, 1988) and that this area encodes space and distance from the observed object (Fogassi *et al.*, 1992, 1999). Given the anatomic–functional connection between the areas F4 and F5 (Luppino *et al.*, 1999), our results can reflect a hypoactivation of a circuit involved in the encoding of the PPS and in transforming object locations into appropriate movements toward them (Colby *et al.*, 1993; Rizzolatti *et al.*, 1997; Duhamel *et al.*, 1998).

Of note, with respect to the source activation in response to participants’ observation of faces being painfully stimulated, we found increased IFG activity in the no-barrier condition. This result aligns with previous findings showing an association between empathy and IFG activation during the observation of facial expressions (Jabbi *et al.*, 2007). Neuroimaging studies have further emphasized the specific role played by the IFG in emotional empathy (Shamay-Tsoory *et al.*, 2009; e.g., emotion recognition: Schulte-Rüther *et al.*, 2007; empathizing with people suffering from a severe threat or harm: Nummenmaa *et al.*, 2008). Additionally, cortical lesions involving the IFG (particularly BA 44 and BA 45) have been shown to be associated with impaired emotional contagion and deficits in emotion recognition (Keysers and Gazzola, 2006).

Overall, this finding suggests that, by preventing the interaction with the observed person, the plexiglass reduced the size of the PPS. This result is aligned with evidence suggesting that PPS representations are highly flexible and change in response to specific experiences and contexts (Di Pellegrino and Làdavas, 2015). In particular, research has shown that the nature of one’s social relationships with others contributes to shaping the PPS (Teneggi *et al.*, 2013). This suggests that, when variables are introduced that alter the space (e.g. a barrier or an unknown individual), the PPS may shrink, as if to create some distance between individuals. By contrast, following a positive social exchange or in the absence of a barrier, the PPS might extend or remap, as if to create a shared space for the interaction (e.g. Berti and Frassinetti, 2000; Serino *et al.*, 2007; Coello *et al.*, 2018; Forsberg *et al.*, 2019).

Finally, the discrepancy between the neural and behavioral levels likely arose because the task used in the study (i.e. judging whether a face was being gently touched or painfully stimulated) was too easy for a major effect to emerge at the accuracy level. By contrast, the neural measure might have been more sensitive to the transparent barrier manipulation. The literature offers several examples of this discrepancy between neural and behavioral results (as previously documented in, e.g., Luck *et al.*, 1996; Heil *et al.*, 2004; Sessa *et al.*, 2014; Schiano Lomoriello *et al.*, 2018, 2022). An alternative explanation for this inconsistency may be that the two selected ERP components and the behavioral measures estimated different aspects of perception. Whereas the early and late ERP components reflected perceptual, cognitive and emotional processing, accuracy and reaction time reflected the entire evaluation process.

To conclude, we would like to discuss a few possible limitations of the present study. Indeed, although faces selected from the ELF database have been used in previous studies to investigate participants’ empathic responses (e.g. Sessa and Meconi, 2015; Schiano Lomoriello *et al.*, 2018; Farmer *et al.*, 2020), it should be considered that they are static images, which may not be the best to mimic an interaction as real as possible. Although this investigation certainly provides an important indication of the relationship between the PPS and empathy, future studies should consider a more ecological design, in terms of both stimuli and potential interactive scenarios. Indeed, if, on the one hand, the paradigm we implemented is a highly controlled one, on the other hand, the potential interaction is still with a stimulus displayed on a computer monitor. Therefore, it will be interesting to implement a new, more ecological task, perhaps in a virtual reality environment. In addition, future studies could investigate the impact of the barrier in social phenomena, such as shared attention, in which the presence of the other has been shown to increase both behaviors, the memory of stimuli (Shteynberg, 2018) and their neural processing (e.g. faces; Schiano Lomoriello *et al.*, 2022). Finally, another aspect that needs to be considered as a limitation of this study is that it lacks the quantification of an actual measure of the PPS. Nonetheless, the present investigation has implications for all situations in which physical barriers are erected to protect individuals by, for example, reducing the risk of spreading disease (i.e. in hospitals and other medical contexts). In this vein, during the COVID-19 pandemic, government restrictions imposed distancing among individuals and implemented strategies such as the use of transparent physical barriers to reduce interpersonal contact, for example, in offices and restaurants. The present results shed light on the implications of such barriers, given their role in reducing empathic neural responses. The findings may be particularly relevant to situations in which empathically connecting with others is crucial—as in health care, psychotherapy and telemedicine. In these contexts, where the empathic resonance between interactive partners is fundamental, awareness of the implications of a transparent barrier for empathy may allow individuals to actively work to minimize this effect.

## Data Availability

Results files and the raw data from the present study are available in the OSF repository at the following link: https://osf.io/6uxgt/.

## Footnotes

1 ∆AIC was computed as the difference in the AIC between the best ranked model and the null model, representing the difference in quality between models.
